# Advanced Brain Tumor Classification in MR Images Using Transfer Learning and Pre-Trained Deep CNN Models

**DOI:** 10.3390/cancers17010121

**Published:** 2025-01-02

**Authors:** Rukiye Disci, Fatih Gurcan, Ahmet Soylu

**Affiliations:** 1 Department of Management Information Systems, Faculty of Economics and Administrative Sciences, Karadeniz Technical University, 61080 Trabzon, Turkey; 2 Department of Computer Science, Faculty of Information Technology and Electrical Engineering, Norwegian University of Science and Technology, 2815 Gjøvik, Norway

**Keywords:** brain tumor classification, deep learning, MR imaging, transfer learning, model performance, clinical diagnostics

## Abstract

Background/Objectives: Brain tumor classification is a crucial task in medical diagnostics, as early and accurate detection can significantly improve patient outcomes. This study investigates the effectiveness of pre-trained deep learning models in classifying brain MRI images into four categories: Glioma, Meningioma, Pituitary, and No Tumor, aiming to enhance the diagnostic process through automation. Methods: A publicly available Brain Tumor MRI dataset containing 7023 images was used in this research. The study employs state-of-the-art pre-trained models, including Xception, MobileNetV2, InceptionV3, ResNet50, VGG16, and DenseNet121, which are fine-tuned using transfer learning, in combination with advanced preprocessing and data augmentation techniques. Transfer learning was applied to fine-tune the models and optimize classification accuracy while minimizing computational requirements, ensuring efficiency in real-world applications. Results: Among the tested models, Xception emerged as the top performer, achieving a weighted accuracy of 98.73% and a weighted F1 score of 95.29%, demonstrating exceptional generalization capabilities. These models proved particularly effective in addressing class imbalances and delivering consistent performance across various evaluation metrics, thus demonstrating their suitability for clinical adoption. However, challenges persist in improving recall for the Glioma and Meningioma categories, and the black-box nature of deep learning models requires further attention to enhance interpretability and trust in medical settings. Conclusions: The findings underscore the transformative potential of deep learning in medical imaging, offering a pathway toward more reliable, scalable, and efficient diagnostic tools. Future research will focus on expanding dataset diversity, improving model explainability, and validating model performance in real-world clinical settings to support the widespread adoption of AI-driven systems in healthcare and ensure their integration into clinical workflows.

## Simple Summary

This study explores the use of pre-trained deep learning models for classifying brain MRI images into four categories: Glioma, Meningioma, Pituitary, and No Tumor. The study uses a publicly available Brain Tumor MRI dataset and applies transfer learning to improve diagnostic accuracy and efficiency by fine-tuning pre-trained models. Xception achieved the highest performance with a weighted accuracy of 98.73%. While the models showed promise in addressing class imbalances, challenges in improving recall for certain tumor types remain. The study highlights the potential of deep learning in transforming medical imaging and clinical diagnostics.

## 1. Introduction

Brain tumors, which result from abnormal cell growth in the brain, are one of the most concerning medical conditions due to their potential severity and impact on a patient’s quality of life. These tumors are generally classified into two categories: primary tumors, which originate within the brain, and secondary tumors, which spread from other parts of the body [1,2,3]. Gliomas and meningiomas are among the most common and dangerous forms of primary brain tumors, with meningiomas being the most prevalent. Gliomas are generally more aggressive, whereas meningiomas are seen more frequently [4,5].

The symptoms of brain tumors can vary widely, ranging from headaches and cognitive impairments to more severe neurological deficits, depending on the tumor’s size, location, and grade [4,6]. Early and accurate diagnosis is critical, as it directly influences the treatment approach and prognosis. However, despite advancements in medical imaging, the diagnosis of brain tumors remains a complex and time-consuming process that often requires specialized expertise and significant waiting times [5,6]. The accurate classification of brain tumors is a critical challenge in the field of medical imaging, as early detection significantly improves patient outcomes [2,3]. Brain tumors, which can range from benign to malignant, present diverse imaging characteristics that often require expert radiological interpretation [4,7,8]. However, the complexity and variability of brain MRI scans make manual diagnosis prone to errors, especially when dealing with large datasets or subtle tumor characteristics [9,10,11]. The rapid advancement of software technologies and digital transformation is revolutionizing healthcare, enabling faster and more accurate diagnoses through AI and deep learning in medical imaging [12,13]. In recent years, artificial intelligence (AI) and deep learning methods have emerged as powerful tools to assist in medical image analysis, offering the potential to enhance diagnostic accuracy, speed, and consistency [7,10,14,15,16].

Deep learning, particularly through the use of convolutional neural networks (CNNs), has demonstrated exceptional performance in various image classification tasks, including medical imaging [4,9,10,17]. CNNs automatically learn hierarchical features from raw image data, making them highly effective for tasks like image segmentation, feature extraction, and classification. In particular, transfer learning has further advanced the field by enabling the reuse of pre-trained models, which are fine-tuned for specific tasks [2,15]. This approach significantly reduces the computational cost and time required to train deep learning models while still achieving high performance [11,18,19]. Early detection is vital for better treatment outcomes, but accurate brain tumor classification remains challenging due to the complexity of medical imaging and large datasets [9,17]. Both traditional diagnostic methods and modern deep learning techniques have shown significant potential in addressing the challenges of brain tumor classification [1,10,20]. While traditional methods rely heavily on expert knowledge and manual interpretation, deep learning offers the advantage of automating the process, potentially reducing human error and improving efficiency [4,7]. As a result, there is an increasing demand for automated systems that can not only classify brain MRI images with high accuracy but also do so in a way that supports clinicians in making timely and informed decisions [7,9,21,22]. These systems hold the promise of improving diagnostic speed, reducing variability, and ultimately enhancing patient outcomes in the field of brain tumor detection [17,20].

The main motivation behind this study is the direct impact early brain tumor diagnosis has on patient treatment and the need to improve this process. The use of modern technologies like deep learning and transfer learning is emphasized as a way to overcome the challenges of traditional methods that rely on manual interpretation and expert knowledge. These automatic classification systems are necessary to reduce human error and increase processing speed. By leveraging advanced deep learning and transfer learning techniques, this study aims to effectively classify brain MRI images, enhancing the accuracy and efficiency of medical imaging systems, and ultimately supporting healthcare professionals in diagnosing brain tumors more effectively. The research will utilize the publicly available Brain Tumor MRI dataset, which includes images categorized into four distinct classes: Pituitary, Meningioma, Glioma, and No Tumor. The study employs state-of-the-art pre-trained deep learning models to assess their performance in brain tumor classification. Through the integration of these cutting-edge techniques, we aim to develop a model capable of providing reliable and scalable brain tumor classifications that can ultimately aid in clinical decision-making. Furthermore, the findings from this study contribute to the growing body of knowledge regarding the application of deep learning in medical image analysis, particularly for brain tumor detection, and open avenues for further research in AI-driven healthcare solutions. The main contributions of this paper are as follows:

- Transfer learning and pre-trained models are employed to classify brain MRI images, aiming for faster, more accurate, and consistent results compared to traditional diagnostic methods.
- Pre-trained models such as Xception, MobileNetV2, InceptionV3, ResNet50, VGG16, and DenseNet121 are utilized through transfer learning to classify brain tumors, reducing training time and computational resources while also enhancing performance and contributing to improved cancer diagnosis.
- The performance of deep learning models, using transfer learning and pre-trained models, is tested on brain tumor MRI data categorized into four distinct classes to evaluate their classification accuracy and overall effectiveness.
- Automated brain tumor classification systems, utilizing transfer learning and pre-trained models, are recommended to provide reliable and scalable models that support clinical decision-making.

The rest of the paper is organized as follows: Section 2 outlines the methodology, detailing the dataset, image preprocessing, and augmentation techniques, along with descriptions of the pre-trained models, model architecture, and fine-tuning process. Section 3 presents the performance analysis, including the results of individual models and their comparative evaluation. Section 4 provides a discussion of the results, including their interpretation, validation of findings, study limitations, and their broader implications in the field. Finally, Section 5 concludes the study and highlights directions for future research in brain tumor classification using deep learning techniques.

## 2. Materials and Methods

**Figure 1:**
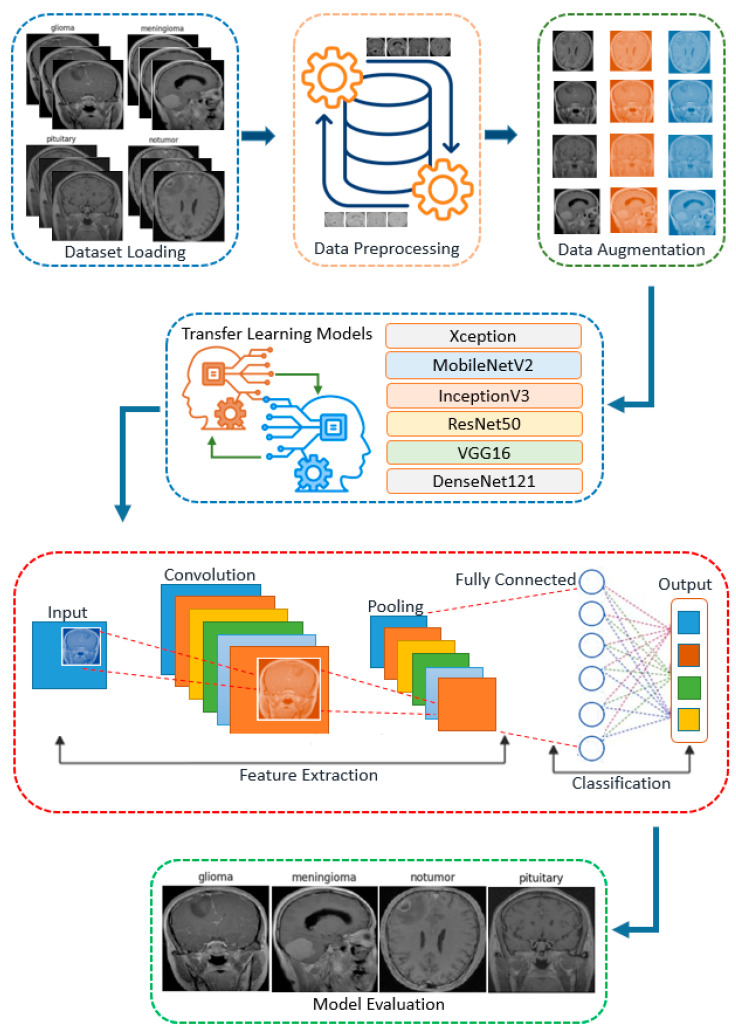
Architecture and workflow of the proposed brain MRI classification methodology.

The methodology employed in this study leverages state-of-the-art deep learning and transfer learning techniques for effective brain MRI classification. This study aims to classify brain MRI images using the publicly available Brain Tumor MRI dataset, which consists of a total of 7023 MRI images spanning four distinct classes: Pituitary, Meningioma, Glioma, and No Tumor. The proposed methodology, as illustrated in Figure 1, provides a comprehensive representation of the architecture and workflow employed in this study, highlighting the integration of deep learning techniques and transfer learning methodologies for the accurate classification of brain MRI images into four distinct classes. 

This process involves key steps such as data description and splitting, data preprocessing, data augmentation, model architecture, fine-tuning, model compilation, and model evaluation, forming a systematic framework designed to achieve accurate and efficient categorization of brain MRI images. As shown in Figure 1, the proposed methodology begins with data preparation, where MRI images are collected, preprocessed, and classified. Then, a pre-trained or specially designed deep learning model is selected, which automatically learns the important features from the images. Using the learned features, the images are classified into different tumor types. Finally, the model’s performance is evaluated using metrics such as the correct classification rate. This sequential framework highlights the integration of data preparation, feature extraction, and evaluation to achieve accurate tumor classification.

### 2.1. Data Description and Splitting

This study utilized the publicly available Brain Tumor MRI dataset, which comprises a total of 7023 MRI images divided into 5712 training images and 1311 testing images [23]. The dataset, publicly accessible on Kaggle, ensures reproducibility and supports the development and validation of classification models [23]. The dataset includes four distinct categories of brain MRI images: pituitary tumors, benign growths in the pituitary gland affecting hormones; meningioma tumors, often benign but pressure-inducing growths from brain coverings; glioma tumors, aggressive cancers from glial cells; and no tumor, normal MRI scans without abnormalities [1,20,23]. The testing set is further distributed with 300 pituitary images, 306 meningioma images, 300 glioma images, and 405 no-tumor images, while the training set includes 1457 pituitary images, 1339 meningioma images, 1321 glioma images, and 1595 no-tumor images [1,23,24]. This dataset provides a balanced distribution of classes, enabling reliable model evaluation and comparison. Additionally, the inclusion of varied tumor types ensures the assessment of classification models under realistic and clinically relevant conditions [25,26].

### 2.2. Data Preprocessing

In this study, several preprocessing and data cleaning steps were applied to the brain tumor MRI images in order to enhance the quality of the data and improve the accuracy and robustness of the deep learning models [1,24,25]. Initially, all images in the dataset were converted to grayscale. This step was necessary to reduce the computational complexity by eliminating the color channels, as the key features for classification are contained in the intensity variations, not in the color information [1,24]. After converting to grayscale, the images were subjected to a blurring process, typically achieved through Gaussian filtering, to reduce high-frequency noise. This preprocessing step helps the model focus on relevant features and minimizes the impact of minor variations and irrelevant details [20,25].

To further enhance the feature extraction process, binary thresholding was applied. This technique transforms the grayscale images into binary images where pixel values above a specified threshold are set to white (foreground), and those below the threshold are set to black (background) [1,17,24]. This highlights the boundaries of the objects (in this case, the tumor regions), making it easier for the model to distinguish between different classes of tumors and healthy tissue. Once the thresholding was applied, the largest contour (i.e., the tumor area) in each image was detected using contour detection algorithms, and the image was cropped to focus on the region of interest (ROI) where the tumor is present [15,22,25]. This helps eliminate unnecessary background and ensures that the model focuses on the relevant parts of the image. After cropping, the images were resized to a standard dimension of 128 × 128 pixels to ensure consistency in input size across all images [20,26].

### 2.3. Data Augmentation

At this stage of the analysis, a number of advanced data augmentation techniques were applied to enhance the diversity of the dataset and strengthen the model’s ability to generalize to unseen data. Augmentation is a method of artificially increasing the size and diversity of the training dataset by applying various transformations [8,20]. This process helps the model become more invariant to changes in image conditions, such as lighting, orientation, and scale. In this study, augmentation included random changes in brightness and contrast. Specifically, brightness was adjusted randomly within the range of 0.8 to 1.2, simulating different lighting conditions under which the MRI images might be captured. This helps the model to adapt to varying illumination during real-world usage. Similarly, contrast values were randomly adjusted in the same range (0.8 to 1.2) to allow the model to learn to identify tumor features under varying contrast levels, which could reflect different imaging settings or equipment [1,18].

After applying the transformations, all augmented images were normalized by dividing the pixel values by 255 to scale them between 0 and 1. This step is crucial for deep learning models as it ensures that the input data are in a consistent numerical range, preventing issues such as exploding or vanishing gradients during training [8]. Normalization accelerates the convergence of the training process and improves the stability of the model. These preprocessing and augmentation techniques were applied to both the training and testing datasets to ensure that the model could learn from a diverse set of input images while maintaining performance across various image qualities and conditions [1,20,26].

### 2.4. Implementation of Pre-Trained CNN Models

In this study, the pre-trained deep learning models employed include Xception, MobileNetV2, InceptionV3, ResNet50, VGG16, and DenseNet121, each representing a state-of-the-art architecture known for its ability to capture complex features and deliver high accuracy in image classification tasks across various domains. Extreme Inception (Xception) is a deep convolutional neural network architecture built upon depthwise separable convolutions, which significantly reduces computational costs while maintaining performance [26,27]. Mobile Neural Network Version 2 (MobileNetV2) is designed for lightweight applications, employing depthwise separable convolutions and inverted residual structures to reduce computational overhead [15,28]. Inception Version 3 (InceptionV3) is a convolutional neural network that incorporates inception modules, allowing it to capture multi-scale features efficiently within a single layer [11,29]. Residual Network 50 (ResNet50) is a 50-layer deep convolutional neural network that introduces residual connections to alleviate the vanishing gradient problem in very deep networks [26,30,31]. Visual Geometry Group 16 (VGG16) is a straightforward deep learning model characterized by its uniform stacking of convolutional layers followed by fully connected layers [1,2,11]. Densely Connected Convolutional Network 121 (DenseNet121) introduces dense connectivity by connecting each layer to every other layer in a feed-forward manner, which reduces redundancy and improves feature reuse [10,20,24,32]. These models have been pre-trained on large-scale datasets, enabling effective transfer learning by fine-tuning them for domain-specific tasks while maintaining their high performance [1,10,31]. The selection of these models was driven by their advanced architectural designs, which include deep convolutional layers, residual connections, and specialized modules, all of which have been empirically proven to excel in complex image classification tasks [1,24,26].

Transfer learning is a machine learning technique where a model leverages knowledge learned from a different but related task to improve learning on a new task. This approach is particularly useful in domains with limited labeled data, as it enables models to use features learned from large, general datasets to enhance performance on specialized tasks. In this study, transfer learning was applied to brain tumor classification by utilizing pre-trained models [18,33]. These models, initially trained on large datasets such as ImageNet, were fine-tuned to the specific task of brain tumor classification. This approach allowed the models to utilize pre-learned weights. Through the application of transfer learning with pre-trained models, the study effectively adapted features learned from large-scale datasets for brain tumor classification [20,25,34].

### 2.5. Model Architecture, Fine-Tuning Process, and Model Compilation

The model architecture designed for this study is structured to effectively learn the complex features of the dataset while improving its ability to generalize to new, unseen data. Table 1 outlines the layer structure and functionality of the proposed model, detailing each component’s role in feature extraction and classification. As shown in the table, the model starts with an input layer that accepts RGB images of size 128 × 128 pixels [2,9,35,36]. This resolution was chosen to balance the computational requirements and the level of detail needed for accurate image classification. The images are then passed through a series of customized layers designed to extract relevant features from the input data. This feature extraction process is critical for identifying the most important patterns in the images, such as tumor characteristics in medical imaging [8,37]. After feature extraction, the model uses a Flatten layer to convert the multidimensional output from the feature extraction layers into a one-dimensional vector. This step is necessary to prepare the features for the fully connected layers, which are responsible for the final classification [3,36,38,39]. The Flatten layer essentially “flattens” the feature map so that it can be fed into the Dense (fully connected) layers, where more complex relationships between features can be learned [36,40].

To mitigate overfitting and enhance the model’s generalization capacity, two dropout layers are introduced. The first dropout layer has a rate of 0.3, and the second one follows with a rate of 0.2 [3,41]. Dropout is a regularization technique that randomly disables a fraction of the neurons during training, forcing the network to rely on different pathways and thus improving its ability to generalize to new data. These dropout layers are critical for preventing the model from memorizing the training data, which could lead to poor performance on unseen data. To further improve the model’s ability to classify brain tumor images accurately, a fully connected (dense) layer with 128 neurons is added. This layer uses the ReLU (Rectified Linear Unit) activation function, which is widely used in neural networks due to its simplicity and effectiveness in avoiding issues like the vanishing gradient problem. ReLU allows the model to capture non-linear relationships between the extracted features, contributing to improved classification performance [36,39]. Finally, the model ends with an Output Layer that has a number of neurons equal to the number of classes (in this case, four brain tumor categories). The Softmax activation function is used here to produce probability scores for each class, ensuring that the model outputs a probability distribution across all possible classes, with the highest probability corresponding to the predicted class [4,36,40,42].

Once the architecture was established, the fine-tuning process began to optimize the model for the specific task of brain tumor classification. During training, the learning rate was set to 0.0001, which was chosen to ensure stable convergence without overshooting the optimal solution [1,26]. The Adam optimization algorithm was used due to its adaptive learning rate properties, which are well-suited for handling complex models and large datasets. Adam is particularly effective in adjusting the learning rate during training, making it a robust choice for deep learning applications. For the loss function, sparse_categorical_crossentropy was selected [17,43]. This is a standard choice for multi-class classification problems where the target labels are integers rather than one-hot encoded vectors. The sparse version of categorical crossentropy is ideal when the labels are provided as integers, as is the case in this study, where each image belongs to one of four tumor classes [1,20,26]. The performance of the model was evaluated using the sparse_categorical_accuracy metric, which tracks the proportion of correct classifications over the total number of predictions. This metric is particularly suitable for multi-class classification problems, as it directly reflects the model’s ability to correctly predict the class for each input image [10,25,44]. Training was conducted using mini batches of 20 images each. Mini-batch gradient descent helps in stabilizing the training process and can lead to faster convergence compared to using the full dataset all at once. The model was trained for 5 epochs, which was found to be sufficient for the model to learn the underlying patterns in the data without overfitting [9,26,41].

In model evaluation, several key metrics are used to assess a model’s performance during both training and testing. Commonly used metrics include accuracy, precision, recall, F1 score, and loss, each offering unique insights into the model’s ability to classify data accurately [21,44]. In addition to these, metrics such as training accuracy, testing accuracy, macro accuracy avg, weighted accuracy avg, training loss, and testing loss are crucial for understanding the model’s generalization and performance across different stages. These metrics collectively help to evaluate how well the model performs on both the training data and unseen test data, providing a comprehensive view of its strengths and weaknesses in tasks like brain MRI classification [4,10,21].

## 3. Experimental Results

In this section, we present and discuss the results obtained from the experiments conducted using six pre-trained deep learning models: Xception, MobileNetV2, InceptionV3, ResNet50, VGG16, and DenseNet121. These models represent various architectures, including convolutional neural networks (CNNs) with different layers and structures. We evaluate their performance using key metrics such as accuracy, precision, recall, F1 score, support, and loss, comparing both training and testing results. In the following subsections, we will first provide an overview of the individual performance metrics for each model to offer a comprehensive understanding of their classification abilities. This will be followed by a detailed comparison across the models, highlighting their relative strengths and weaknesses, as well as their potential for deployment in medical image analysis tasks. Finally, we will discuss the implications of these findings and their potential impact on medical image analysis.

### 3.1. Xception

The Xception model demonstrated exceptional performance in the classification of brain MRI images, as summarized in Table 2 and illustrated by the confusion matrix in Section 3.7, which highlights its classification performance across four classes. The metrics include accuracy, precision, recall, F1 score, and support, offering a clear understanding of the model’s strengths and weaknesses across different classes. The training accuracy of 0.9952, testing accuracy of 0.9527, and weighted accuracy average of 0.9873 demonstrate strong generalization, though the higher training accuracy indicates a slight overfitting tendency. The training loss of 0.0193 is significantly lower than the testing loss of 0.1214, further supporting this observation. The macro averages for precision, recall, and F1 score are 0.9517, 0.9486, and 0.9491, respectively, indicating balanced performance across all classes. The weighted averages, which take into account the class sizes, show slightly higher values, emphasizing the model’s ability to handle class imbalances effectively. On a class-by-class basis, the results indicate strong overall performance, with the No Tumor class achieving the highest metrics. Its precision of 0.9975, recall of 1.0000, and F1 score of 0.9988 demonstrate the model’s ability to accurately identify and avoid misclassifying cases in this category. On the other hand, the Glioma class shows slightly lower recall at 0.8867, indicating that some true cases are missed. The Meningioma class has a precision of 0.8724, which suggests more false positives in this category compared to the others. The Pituitary class performs consistently well with an F1 score of 0.9562, reflecting a balanced trade-off between precision and recall. In conclusion, the Xception model achieves excellent classification performance, particularly for the No Tumor and Pituitary categories, while leaving room for improvement in the Glioma and Meningioma classes. These results highlight the model’s reliability and potential for medical image analysis applications.

### 3.2. MobileNetV2

The MobileNetV2 model demonstrated strong performance in the classification of brain MRI images, as summarized in Table 3 and illustrated by the confusion matrix in Section 3.7, which highlights its classification performance across four classes. The metrics include accuracy, precision, recall, F1 score, and support, offering a clear perspective on the model’s strengths and areas for improvement. The training accuracy of 0.9898 and testing accuracy of 0.9451 suggest good generalization, whereas the weighted accuracy average of 0.9815 reflects the model’s strong performance across both training and testing samples. The training loss of 0.0259 compared to the testing loss of 0.1398 further supports this observation. The macro averages for precision, recall, and F1 score are 0.9426, 0.9404, and 0.9411, respectively, indicating balanced performance across all classes. The weighted averages, which account for class imbalances, show slightly higher values, with an F1 score of 0.9457. These results confirm the model’s ability to handle datasets with uneven distributions effectively. When analyzing performance by class, the No Tumor category stands out with the highest metrics, achieving a precision of 0.9893, recall of 1.0000, and an F1 score of 1.0000, showcasing the model’s excellent ability to classify this class accurately. The Pituitary class also performs well, with an F1 score of 0.9552, though its recall of 0.9300 suggests occasional missed cases. The Glioma class shows balanced results with an F1 score of 0.9094, but its recall of 0.9067 indicates some true positives are not detected. Likewise, the Meningioma class achieves an F1 score of 0.8999, with a slightly lower precision of 0.8761, indicating more false positives in this class. In conclusion, the MobileNetV2 model delivers strong overall performance, excelling in certain classes while leaving room for improvement in others.

### 3.3. InceptionV3

The InceptionV3 model demonstrated strong performance in classifying brain MRI images, as summarized in Table 4 and illustrated by the confusion matrix in Section 3.7. The model’s performance across four classes—Glioma, Meningioma, No Tumor, and Pituitary—is evaluated using metrics such as accuracy, precision, recall, F1 score, and support, providing a comprehensive understanding of its strengths and weaknesses. The training accuracy of 0.9810 and testing accuracy of 0.9451 suggest that the model generalizes well, although the difference between these values hints at a slight overfitting tendency.

The weighted accuracy average of 0.9743 further reflects the model’s consistent performance across both training and testing samples. Additionally, the training loss of 0.0568 compared to the testing loss of 0.1386 supports the idea that the model may be overfitting slightly, as indicated by the higher testing loss. The macro averages for precision, recall, and F1 score are around 0.941, indicating balanced performance, while the weighted averages (F1 score of 0.9452) reflect the model’s ability to handle class imbalances. When analyzing performance by class, the No Tumor category shows the best results, with a precision of 1.0000, recall of 0.9926, and an F1 score of 0.9963, showcasing the model’s high accuracy in identifying this class. The Pituitary class also performs well with an F1 score of 0.9567, though its recall of 0.9500 indicates a few missed cases. The Glioma class demonstrates balanced performance with an F1 score of 0.9129, but its slightly lower recall of 0.9433 suggests some true positives are not identified. Similarly, the Meningioma class achieves an F1 score of 0.8978, with a precision of 0.9180, but its recall of 0.8784 points to some false negatives in this category. As a result, the InceptionV3 model delivers strong overall performance, particularly excelling in the No Tumor category, while leaving room for improvement in the Glioma and Meningioma classes.

### 3.4. ResNet50

The ResNet50 model demonstrated solid performance in classifying brain MRI images, as summarized in Table 5 and depicted in the confusion matrix in Section 3.7. The model’s performance across four classes is evaluated using various metrics such as accuracy, precision, recall, F1 score, and support, providing insights into its strengths and limitations. The training accuracy of 0.9897 and testing accuracy of 0.9062 suggest that the model generalizes reasonably well, though the noticeable gap between these values indicates some overfitting. The weighted accuracy average of 0.9741 reflects consistent performance across both training and testing samples. Additionally, the training loss of 0.0273 compared to the higher testing loss of 0.2336 reinforces the likelihood of overfitting, as suggested by the increased testing loss. The macro averages for precision, recall, and F1 score are 0.9178, 0.8993, and 0.9010, respectively, indicating reasonably balanced performance across all classes. The weighted averages (F1 score of 0.9045) show slightly higher values, demonstrating the model’s effectiveness in managing class imbalances. When analyzing performance by class, the No Tumor category performs excellently with a precision of 0.9056, recall of 0.9951, and an F1 score of 0.9482, indicating the model’s high accuracy in identifying this class. The Pituitary class also delivers strong results with an F1 score of 0.9301, although its recall of 0.8867 suggests a few missed cases. The Glioma class performs well, with an F1 score of 0.8825, but its slightly lower recall of 0.9767 indicates that some true positives are not detected.

### 3.5. VGG16

The VGG16 model demonstrated strong performance in classifying brain MRI images, as summarized in Table 6 and illustrated by the confusion matrix in Section 3.7. The model’s performance is assessed across four classes using metrics like accuracy, precision, recall, F1 score, and support, offering a comprehensive overview of its effectiveness. The training accuracy of 0.9721 and testing accuracy of 0.9504 suggest that the model generalizes well, with only a slight gap indicating minimal overfitting. The weighted accuracy average of 0.9680 further highlights consistent performance across both training and testing samples. Additionally, the training loss of 0.0762 and testing loss of 0.1270 indicate a reasonable model fit, with only a slight increase in the testing loss. The macro averages for precision, recall, and F1 score are 0.9477, 0.9470, and 0.9470, respectively, reflecting balanced performance across all classes. The weighted averages (F1 score of 0.9478) show similar values, suggesting that the model effectively manages class imbalances. When analyzing performance by class, the No Tumor category stands out with the highest metrics, achieving a precision of 1.0000, recall of 0.9901, and an F1 score of 0.9950, showcasing the model’s ability to accurately classify this class. The Pituitary class also performs well with an F1 score of 0.9571, though its recall of 0.9367 indicates occasional missed cases. The Glioma class shows strong results, with an F1 score of 0.9231 and a recall of 0.9200, indicating effective classification with few false negatives.

### 3.6. DenseNet121

The DenseNet121 model demonstrated solid performance in classifying brain MRI images, as summarized in Table 7 and illustrated by the confusion matrix in Section 3.7. The model’s performance is evaluated across four classes using metrics such as accuracy, precision, recall, F1 score, and support, providing insights into its strengths and weaknesses. The training accuracy of 0.9652 and testing accuracy of 0.9285 suggest that the model generalizes well, though the slight gap between these values indicates a small difference in performance. The weighted accuracy average of 0.9583 further reflects consistent performance across both training and testing samples.

Additionally, the training loss of 0.0878 compared to the higher testing loss of 0.1798 reinforces the possibility of overfitting, as indicated by the increased testing loss. The macro averages for precision, recall, and F1 score are 0.9244, 0.9230, and 0.9220, respectively, showing balanced performance across all classes. The weighted averages (F1 score of 0.9265) demonstrate the model’s ability to handle class imbalances effectively. When analyzing performance by class, the No Tumor category performs exceptionally well with a precision of 0.9643, recall of 1.0000, and an F1 score of 0.9818, indicating high accuracy in identifying this class. The Pituitary class also delivers strong results with an F1 score of 0.9483, though its recall of 0.9920 suggests that the model occasionally misses some cases.

### 3.7. Overall Comparison of Model Performances

**Figure 2:**
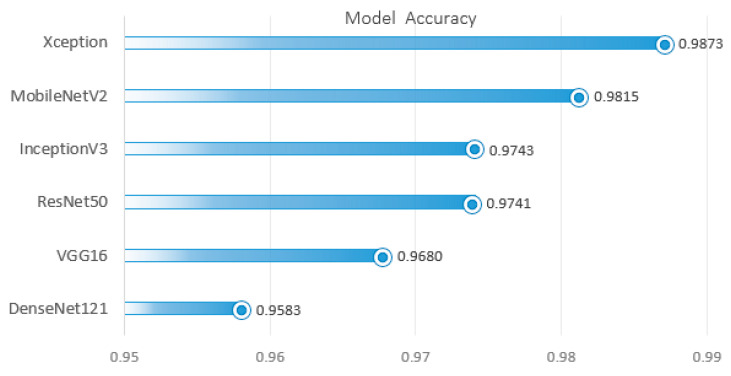
Overall performance comparison of the implemented models with weighted mean accuracy.

**Figure 3:**
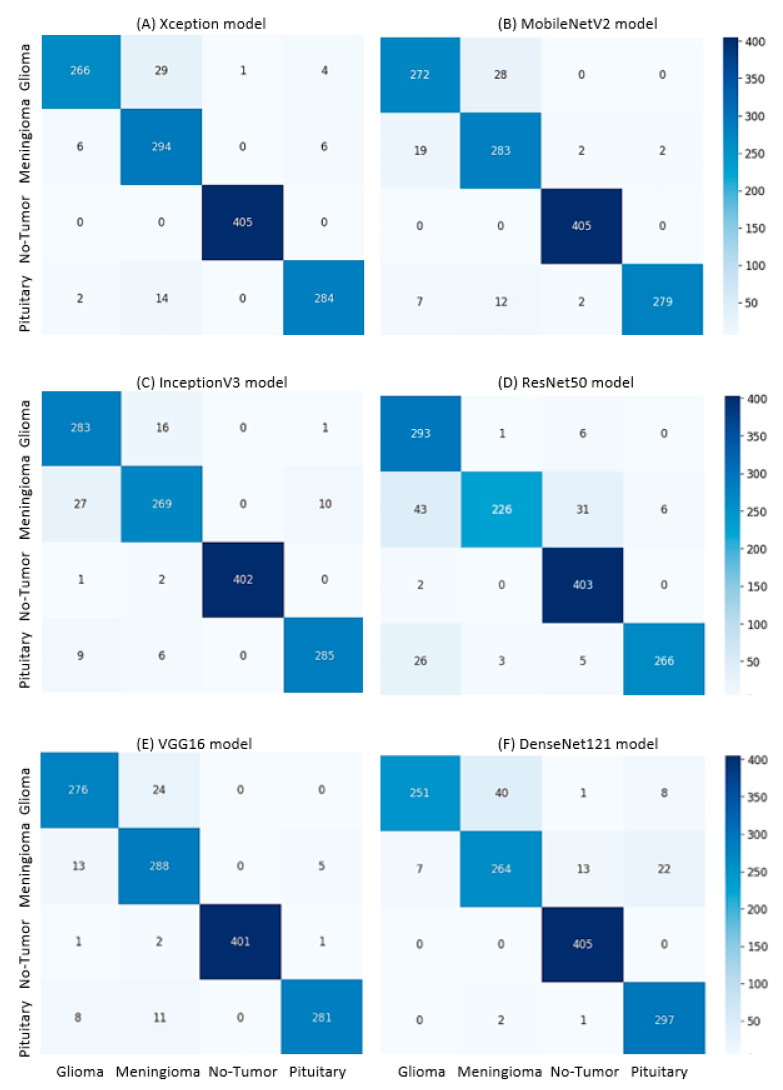
Confusion matrices of pre-trained models across four tumor categories: (**A**) Xception, (**B**) MobileNetV2, (**C**) InceptionV3, (**D**) ResNet50, (**E**) VGG16, and (**F**) DenseNet121.

The pre-trained models used in this study were evaluated using several metrics for tumor classification, and their performance was compared to assess their effectiveness in brain tumor detection. Table 8 provides a comparison of the performance metrics of all the implemented models, ranked according to the Weighted Accuracy Avg, from highest to lowest. Figure 2 illustrates the performance of all the implemented models, with a focus on the Weighted Accuracy Avg. The models are ranked from highest to lowest based on this metric (see Figure 2), highlighting their ability to perform consistently across both training and testing sample sizes. Additionally, Figure 3 presents the confusion matrices of six pre-trained models across four tumor categories, providing a detailed comparison of their classification performance.

The Xception model stands out with the highest weighted accuracy of 0.9873, followed closely by MobileNetV2 at 0.9815 and InceptionV3 at 0.9743 (see Figure 2). These top-performing models demonstrate strong generalization with high testing accuracies, while also maintaining low training losses, indicating minimal overfitting. The ResNet50 model, with a weighted accuracy of 0.9741, ranks just below InceptionV3, but shows a noticeable gap in testing accuracy (0.9062) and higher testing loss (0.2336), suggesting more significant overfitting compared to the top models (see Table 8). VGG16 follows with a weighted accuracy of 0.9680, demonstrating solid performance overall, while DenseNet121, despite performing well with a weighted accuracy of 0.9583, ranks lowest in this comparison (see Table 8 and Figure 2). DenseNet121 also exhibits a higher testing loss (0.1798), indicating less effective generalization than the other models. Overall, while Xception, MobileNetV2, and InceptionV3 lead in performance, all the models show varying levels of effectiveness, with specific areas for improvement, particularly in recall and precision for certain classes.

## 4. Discussion, Validity, and Limitations

This study evaluates the performance of six state-of-the-art pre-trained models for brain MRI classification and demonstrates their effectiveness in handling the complexities of this task. Among these, Xception emerged as the top-performing model, with a weighted accuracy of 98.73%, demonstrating strong generalization and robustness. The results of this study align with prior research, which consistently highlights the strengths of pre-trained CNNs in medical image analysis [7,20,24,26,45,46]. Pre-trained models like U-Net, InceptionV3, Xception, MobileNetV2, ResNet50, VGG16, DenseNet121, UNet++, and SegNet have been widely utilized in previous brain tumor studies, with transfer learning showing promising results across various dataset settings and demonstrating strong performance in tumor detection tasks [11,20,24,25,33,47]. However, the performance disparities across tumor categories, particularly in Glioma and Meningioma, underscore the need for advanced techniques to handle subtle and overlapping imaging features [8,26]. While Xception and MobileNetV2 excel in computational efficiency, emerging pre-trained models such as EfficientNet, Vision Transformers (ViT), Swin Transformer, ConvNeXt, and DeiT show significant promise for enhancing performance in tumor detection and clinical applications [17,21,48,49].

Classical deep CNNs, such as those used in [50,51], achieved accuracies of 96.3% and 96.56%, respectively. While these models demonstrate the potential of deep learning for medical imaging, their simpler architectures and lack of advanced design components limit their capacity to capture hierarchical features effectively. Traditional approaches like SVM in [52] achieved 97.1% accuracy, demonstrating moderate success. Models such as AlexNet and VGG16, used in [53,54,55], achieved accuracies ranging from 98.69% to 99.04%. Hybrid models, such as [ResNet18 + ShallowNet] + SVM in [56], achieved 98.02% accuracy. Similarly, ensemble approaches in [57], combining multiple models like ResNet and AlexNet, reached 99.30%. These methods benefit from combining complementary strengths but often come with increased computational complexity. EfficientNet, noted for its parameter efficiency, achieved 98.86% accuracy in [58]. Deep CNN models achieving the highest classification accuracies, such as Dense Efficient-Net (99.97%) [59], DeepTumorNet (99.67%) [60], TumorResNet (99.33%) [47], Hybrid MobileNetV2 (99.92%) [61], and Hybrid GoogLeNet (99.10%) [62], highlight the superior performance of advanced architectures in optimizing classification outcomes for complex medical imaging tasks. TumorGANet, which achieved an accuracy of 99.53% (see [63]), demonstrates exceptional performance in tumor detection.

The validity of this study is demonstrated through the systematic design and rigorous evaluation of pre-trained deep learning models for brain MRI classification. The use of a publicly available Brain Tumor MRI dataset ensured transparency and reproducibility [1,20,23,26]. The use of a balanced Brain Tumor MRI dataset, representing four distinct classes (Glioma, Meningioma, Pituitary, and No Tumor), ensured that the models were trained and tested on data with equitable class distribution, reducing the risk of biased predictions [24,25,38,57]. Multiple metrics, including accuracy, precision, recall, F1 score, and loss, were used to validate model performance comprehensively. These metrics not only assessed the overall accuracy but also highlighted the models’ ability to handle class imbalances and predict minority classes accurately [1,25,26]. For example, the highest-performing model, Xception, demonstrated a testing accuracy of 95.27% and a weighted F1 score of 95.29%, indicating strong generalization. Advanced data augmentation techniques (e.g., random changes in brightness, contrast, and orientation) were applied to simulate real-world variations, improving model robustness and generalization [8,14,64]. Preprocessing steps like resizing, normalization, and region-of-interest extraction further enhanced data quality and performance. The study validated its findings through a comparative analysis of six state-of-the-art pre-trained models, demonstrating consistent performance and reliability [1,26,33,56]. Transfer learning, dropout regularization, and fine-tuning helped achieve high classification accuracy and reduce overfitting. For example, the Xception model showed minimal performance gaps (training accuracy: 99.52%, testing accuracy: 95.27%). Transfer learning reduced training time and highlighted the scalability of pre-trained models for medical imaging tasks [8,20]. Confusion matrices provided insights into model performance, with high precision and recall for the “No Tumor” and “Pituitary” categories, confirming the models’ reliability in diagnosing critical tumor types [33,44,59,65].

Despite its strengths, the study has several limitations. While the dataset was balanced, its diversity was limited, lacking variations in MRI protocols, scanner types, and patient demographics. Expanding the dataset to include cases from different populations and centers would improve model robustness in diverse clinical environments [7,60]. Leveraging big data from multi-center collaborations and integrating virtual reality technologies for immersive visualization of medical images could enhance model training, interpretability, and clinical adoption, providing a more comprehensive approach to tumor detection and diagnosis [3,45,66,67]. The models showed class-specific weaknesses, especially in recall for Glioma and Meningioma, suggesting missed true positives [7,65]. Advanced techniques like class-specific fine-tuning or ensemble methods could address these issues [58,68]. Additionally, the lack of external validation and cross-validation limits generalizability, highlighting the need for further evaluation in real-world settings. The black-box nature of deep learning models poses challenges for clinical adoption, where explainability is crucial. Techniques like saliency maps or attention mechanisms could improve interpretability [8,58,69]. Finally, the computational demands of deploying these models, especially in resource-constrained environments, present practical challenges. Streamlining models for low-resource hardware could improve accessibility. Addressing these limitations will enhance the models’ reliability and applicability in real-world medical imaging, ultimately improving patient outcomes.

## 5. Conclusions

This study aimed to enhance the classification of brain MRI images using state-of-the-art pre-trained deep learning models, focusing on Xception, MobileNetV2, InceptionV3, ResNet50, VGG16, and DenseNet121. The results demonstrate that advanced transfer learning techniques can effectively classify brain tumors, providing high accuracy and balanced performance across multiple metrics. Among the tested models, Xception emerged as the top performer, achieving a weighted accuracy of 98.73% and consistently demonstrating high generalization with minimal overfitting. Similarly, MobileNetV2 and InceptionV3 delivered robust results, with weighted accuracies exceeding 97%, showcasing their ability to handle complex classification tasks effectively.

These findings underline the potential of deep learning techniques in automating and enhancing the diagnostic process, supporting clinicians in making more timely and accurate decisions. Future research should focus on addressing class-specific performance disparities by incorporating more diverse datasets and advanced techniques such as ensemble learning or hybrid models. Additionally, exploring interpretability and explainability methods could provide clinicians with greater confidence in integrating these systems into routine medical practice. Future studies could explore emerging pre-trained models such as Vision Transformers (ViT), Swin Transformer, ConvNeXt, Data-efficient Image Transformer (DeiT), and newer approaches like Segment Anything Model (SAM) and MixVision Transformers (MiT), which show significant promise for enhancing performance in tumor detection and advancing clinical applications. The outcomes of this research contribute to the growing field of AI-driven healthcare solutions, emphasizing their transformative potential in medical imaging and diagnostics.

## Figures and Tables

**Table 1:** Layer-wise description of the proposed model architecture.

Layer	Description
1. Input Layer	The model starts with an input layer that accepts images of size IMAGE_SIZE × IMAGE_SIZE × 3. This represents RGB images.
2. Base Model	A pre-trained model (e.g., Xception, ResNet50, etc.) used for feature extraction. It contains pre-trained weights from large datasets.
3. Flatten	This layer flattens the multidimensional output from the base model into a one-dimensional vector, making it suitable for fully connected layers.
4. Dropout (0.3)	A regularization layer that randomly drops 30% of the neurons during training to prevent overfitting and encourage the model to generalize better.
5. Dense (128, ReLU)	A fully connected layer with 128 neurons and ReLU activation. It learns complex features and patterns from the flattened features.
6. Dropout (0.2)	Another regularization layer, this time dropping 20% of the neurons to further prevent overfitting during training.
7. Dense (4, Softmax)	The output layer, with neurons equal to the number of unique classes. Output layer with 4 neurons (for 4 classes) and Softmax activation.

**Table 2:** Classification metrics of Xception model.

Model	Class	Precision	Recall	F1 Score	Support
Xception	Glioma	0.9708	0.8867	0.9268	300
	Meningioma	0.8724	0.9608	0.9145	306
	No Tumor	0.9975	1.0000	0.9988	405
	Pituitary	0.9660	0.9467	0.9562	300
	Testing Macro Avg	0.9517	0.9486	0.9491	1311
	Testing Weighted Avg	0.9550	0.9527	0.9529	1311
	Training Accuracy	0.9952			5712
	Testing Accuracy	0.9527			1311
	Macro Accuracy Avg	0.9740			7023
	Weighted Accuracy Avg	0.9873			7023
	Training Loss	0.0193			5712
	Testing Loss	0.1214			1311

**Table 3:** Classification metrics of MobileNetV2 model.

Model	Class	Precision	Recall	F1 Score	Support
MobileNetV2	Glioma	0.9134	0.9067	0.9094	300
	Meningioma	0.8761	0.9248	0.8999	306
	No Tumor	0.9893	1.0000	1.0000	405
	Pituitary	0.9915	0.9300	0.9552	300
	Testing Macro Avg	0.9426	0.9404	0.9411	1311
	Testing Weighted Avg	0.9460	0.9451	0.9457	1311
	Training Accuracy	0.9898			5712
	Testing Accuracy	0.9451			1311
	Macro Accuracy Avg	0.9675			7023
	Weighted Accuracy Avg	0.9815			7023
	Training Loss	0.0259			5712
	Testing Loss	0.1398			1311

**Table 4:** Classification metrics of InceptionV3 model.

Model	Class	Precision	Recall	F1 Score	Support
InceptionV3	Glioma	0.8844	0.9433	0.9129	300
	Meningioma	0.9180	0.8784	0.8978	306
	No Tumor	1.0000	0.9926	0.9963	405
	Pituitary	0.9635	0.9500	0.9567	300
	Testing Macro Avg	0.9415	0.9411	0.9409	1311
	Testing Weighted Avg	0.9461	0.9449	0.9452	1311
	Training Accuracy	0.9810			5712
	Testing Accuracy	0.9451			1311
	Macro Accuracy Avg	0.9631			7023
	Weighted Accuracy Avg	0.9743			7023
	Training Loss	0.0568			5712
	Testing Loss	0.1386			1311

**Table 5:** Classification metrics of ResNet50 model.

Model	Class	Precision	Recall	F1 Score	Support
ResNet50	Glioma	0.8049	0.9767	0.8825	300
	Meningioma	0.9826	0.7386	0.8433	306
	No Tumor	0.9056	0.9951	0.9482	405
	Pituitary	0.9779	0.8867	0.9301	300
	Testing Macro Avg	0.9178	0.8993	0.9010	1311
	Testing Weighted Avg	0.9171	0.9062	0.9045	1311
	Training Accuracy	0.9897			5712
	Testing Accuracy	0.9062			1311
	Macro Accuracy Avg	0.9480			7023
	Weighted Accuracy Avg	0.9741			7023
	Training Loss	0.0273			5712
	Testing Loss	0.2336			1311

**Table 6:** Classification metrics of VGG16 model.

Model	Class	Precision	Recall	F1 Score	Support
VGG16	Glioma	0.9262	0.9200	0.9231	300
	Meningioma	0.8862	0.9412	0.9129	306
	No Tumor	1.0000	0.9901	0.9950	405
	Pituitary	0.9785	0.9367	0.9571	300
	Macro Avg	0.9477	0.9470	0.9470	1311
	Weighted Avg	0.9518	0.9476	0.9478	1311
	Training Accuracy	0.9721			5712
	Testing Accuracy	0.9504			1311
	Macro Accuracy Avg	0.9613			7023
	Weighted Accuracy Avg	0.9680			7023
	Training Loss	0.0762			5712
	Testing Loss	0.1270			1311

**Table 7:** Classification metrics of DenseNet121 model.

Model	Class	Precision	Recall	F1 Score	Support
DenseNet121	Glioma	0.9660	0.8370	0.8969	300
	Meningioma	0.8590	0.8630	0.8610	306
	No Tumor	0.9643	1.0000	0.9818	405
	Pituitary	0.9083	0.9920	0.9483	300
	Macro Avg	0.9244	0.9230	0.9220	1311
	Weighted Avg	0.9273	0.9289	0.9265	1311
	Training Accuracy	0.9652			5712
	Testing Accuracy	0.9285			1311
	Macro Accuracy Avg	0.9469			7023
	Weighted Accuracy Avg	0.9583			7023
	Training Loss	0.0878			5712
	Testing Loss	0.1798			1311

**Table 8:** Comparison of performance metrics of all implemented models.

Model	Training Accuracy	Testing Accuracy	Macro Accuracy Avg	Weighted Accuracy Avg	Training Loss	Testing Loss
Xception	0.9952	0.9527	0.9740	0.9873	0.0193	0.1214
MobileNetV2	0.9898	0.9451	0.9675	0.9815	0.0259	0.1398
InceptionV3	0.9810	0.9451	0.9631	0.9743	0.0568	0.1386
ResNet50	0.9897	0.9062	0.9480	0.9741	0.0273	0.2336
VGG16	0.9721	0.9504	0.9613	0.9680	0.0762	0.1270
DenseNet121	0.9652	0.9285	0.9469	0.9583	0.0878	0.1798

## Data Availability

The datasets used in this study are publicly available, and access links are provided as references in the dataset section of the article.
